# An evaluation of deep-forehead temperature (spoton^®^) in ICU patients after cardiac surgery

**DOI:** 10.1186/2197-425X-3-S1-A111

**Published:** 2015-10-01

**Authors:** H Kato, S Kawashima, S Mimuro, Y Obata, M Doi, Y Nakajima

**Affiliations:** Department of Anesthesia and Intensive Care, Hamamatsu University School of Medicine, Hamamatsu, Japan

## Introduction

Core temperature is important for the safety of patients in ICU. Although blood temperature (Tbl) has been accepted as the gold standard for core temperature monitoring, it requires an invasive procedure. A recently developed new thermometer, SpotOn^®^(3M, St. Paul, MN) that measures deep-forehead temperature (Tdf) can measure core temperature during surgery1) in a noninvasive manner. However, its accuracy in ICU patients is not known.

## Objectives

The purpose of this study is to compare the accuracy of Tdf with pulmonary artery blood temperature (Tpa) in ICU patients. In addition, we compared the accuracies of Tdf and Tbl to determine which value is closer to Tpa.

## Methods

We studied 20 postoperative cardiac surgery patients. To monitor their core temperature, we measured Tpa using a pulmonary artery thermistor catheter. We used SpotOn^®^ and a urinary thermistor catheter to measure Tdf and Tbl, respectively. All temperatures were recorded at 1-min intervals after patients' admission to ICU; temperature recording continued until the pulmonary artery catheter was removed. We considered Tpa as the reference value and compared Tdf and Tbl with Tpa using Bland-Altman analyses. We determined an accuracy of 0.5°C to be clinically acceptable. The differences between Tdf/Tbl and Tpa were analyzed using paired Student's t-test. A p-value of < 0.05 was considered statistically significant.

## Results

Among the 20 patients, 16 were males and 4 were females (mean age, 66.0 years, range 45-78 years; mean weight 56.2 kg, range 43-66.7 kg; mean height 159.5 cm, range 117.2-178.0 cm). The mean duration of measurement was 865 min (range, 251-2283 min). A total of 16407 value points were analyzed. Tpa ranged from 35.6°C to 39.2°C. The mean average difference between Tdf and Tpa (i.e., Tdf minus Tpa) was −0.28°C (95% limits of agreement: ± 0.88); 79.0% of the differences were ≤0.5°C. The mean average difference between Tbl and Tpa was 0.04°C (95% limits of agreement: ± 0.60); 94.5% of the differences were ≤0.5°C. The difference between Tbl and Tpa was significantly smaller than the difference between Tdf and Tpa (p = 0.000).

## Conclusions

We conclude that Tdf has clinically sufficient accuracy but its accuracy is inferior to that of Tbl.
